# Reply to Editorial, May, 1898

**Published:** 1898-08

**Authors:** G. Carleton Brown


### Domestic Correspondence.

### REPLY TO EDITORIAL, MAY, 1898.

To the Editor:

Sir,—Owing to your long-standing and outspoken antagonism to dental examining boards, and all protective legislation of whatever kind, your editorial in the May issue of your journal was not a surprise, as you always have at least been consistent in maintaining that all the knowledge contained in the dental profession was centred in the heads of the professors of the dental colleges, and of course more particularly those holding chairs in a certain institution.

That there could possibly exist men in the mere every-day walk of the profession who could know enough to examine the graduates of a college has been denied by you time and again, so the animus of your article is too apparent to need much attention. You demand the repeal of the obnoxious law, and say that, "It is deeply to be regretted that the dentists of New Jersey have had this scandalous piece of work thrust upon them, for we are willing to believe that all are not responsible. As it stands at present, New Jersey has put back dental education in that State to where it was prior to the organization of the National Association of Dental Faculties, and has undone within its borders the work of fourteen years." You, however, fail to state how this law undoes the work of fourteen years, the only point, in fact, that you attempt to make against the law being the clause which allows an application for examination to be made by a person not a graduate of a dental school upon the written recommendation of at least five licensed dentists of this State of five years standing, vouching for his qualification to take such examination.

It is a simple matter to prove that your deductions are false, and not in accord with those of other prominent teachers. Permit me to call your attention to portions of a discussion on a paper read by Dr. Jack before the Odontological Society of Pennsylvania, on November 13, 1892, entitled, "Should Examining Boards have Power to grant Certificates of Qualification to Undergraduates?" I think this will prove interesting reading in connection with certain utterances of yourself and the other gentlemen who participated in that discussion, a report of which was, I think, published in your journal, and which was afterwards published officially in book form by the Society. I quote from the book.

In his article Dr. Jack treats the subject in his usual broad and comprehensive way, and the discussion is opened by Dr. Peirce.

"*Dr. Peirce.*—I rather hesitate to express my views on this subject, as it is a matter of some delicacy, inasmuch as I am connected with a school, and it may be somewhat warped with my associations. There is one thing we need to remember, and that is not to confuse incompetent examining boards with competent ones.

"My views are that competent examining boards should be thoroughly prepared to examine and say whether the applicant is qualified to practise dentistry. If we have these, they are for the purpose of judging of the qualifications of applicants. They have no right in my estimation, appointed as they are by the Legislature, to ask the applicant whether he has a diploma, or where he got his education. All they want to do is to ascertain if the applicant is qualified to practise dentistry, and it matters not where that qualification is obtained, so long as he possesses it. While I have that feeling, still I concur in the sentiments of the paper as to disqualification of applicants who have come up before the board; yet the fact still remains that the law has no right to ask the question where the information was gained.

"*Dr. Kirk.*—This is one of the subjects, I must confess, in which I have only a limited interest. This centres so much in other directions that I have not sufficient to go round.

"If I understand correctly the statements made on this subject, that in the effort to obtain State legislation regulating the practice of dentistry, the committees have been met by the problem, the relation of State examining boards to college faculties. The Constitution of the United States and the laws of the various States are repugnant to anything that looks like class legislation, and a law which would prohibit any man from practising dentistry, unless he was a regular graduate of a dental institution, would savor of that idea. Dr. Peirce has very clearly shown that when he states that such legislation would inhibit the class who wish to become self-educated. As a matter of principle, I do not see any objection to saying whether a man should not qualify himself to practise dentistry. The question is, Can he do it? It is the function of the State examining boards to answer that question. . . .

"The State examination, if it is properly conducted, should be the barrier to the entrance of incompetent men into dentistry. Whether we should or should not examine men who have not passed a college examination, it seems to me, hinges on that point of the law in respect to class legislation.

"*Dr. Guilford.*—The remarks of Dr. Peirce pleased me very much, and he covered, I think, the ground very thoroughly.

"*Dr. Truman.*—I find very little in this question. I have been astonished at the first and last speech. Here is a dean who is not satisfied with the teaching of his own school. He wants the diploma discredited by having the board re-examine the students. It is astonishing to me. I do not care to have any board examine a graduate of the University of Pennsylvania. I question their ability to do it. I am opposed to this granting of certificates, and I am surprised that my friend, Dr. Guilford, should defend a practice that will certainly end in discrediting dental education. It would be better to return to the old plan of every man studying in private and securing a certificate from his preceptor. The majority of boards may or may not be good."

It seems to me that here we have a very careful handling of the subject by some of our most noted teachers, and it only seems to be necessary to point out the fact that it would be practically impossible for an unworthy man to procure the necessary credentials, and if he could not pass the examinations, he, of course, would not be licensed. But it might happen that an old practitioner—one thoroughly qualified and competent in every respect, still not a graduate of a dental school, but perhaps an M.D., or a teacher himself—might wish to remove to this State; this clause in our law would allow us to accept him, without which it would be impossible.

There is one charge against the New Jersey Board which you make that really has no bearing on the present subject, but which is so misleading that it should be exposed. You say, "The most persistent State in the Union to demand, through its board, a higher standard has been New Jersey; indeed, this board has recently declared it would not recognize any college that failed to come up to a standard far higher than that adopted by the National Association of Dental Faculties at its last meeting."

The standard for admission to the recognized list of New Jersey is that established by the National Association of Dental Examiners and, consequently, that of all States that are members of the Association. Whether they all live up to them or not is another matter. In New Jersey, if we agree to live up to a certain rule or line of conduct, we do our best to carry out our agreement, regardless of the fact that some one may be hurt, who has, perhaps, been sailing under false colors, much to his own profit.

In the last paragraph of your editorial you not only ask for the repeal of the law, but say, "We have no doubt but that the best thinking men of New Jersey will take measures to effect this, for we are confident that there is a large body there who will not rest until this is accomplished." Now, it will probably be very gratifying to you to know—if you are not already acquainted with the fact—that there has been an association formed for the express purpose of fighting the law. It is composed of the owners, managers, assistants, students, and backers of the various "dental associations," "dental companies," "dental parlors," etc., throughout the State, some of whom are regularly licensed, but more of whom are not, who were, however, able, under the old law, to cover their actions by certain defects and deficiencies in its construction. These have, however, been remedied in the new law, and the breakers of the law and their abettors, brought to bay at last, are now doing the very thing which you advise in your editorial. This does not seem so strange, however, when we remember that back in 1890 this same class of professional (?) men and certain colleges of New York and Philadelphia were the only ones to oppose the bill then before the Legislature.

I congratulate you on the renewal of old associations.

Yours truly,

G. Carleton Brown.

